# Spatial Distribution and Local Coordination Regulation of Nano-Oxide-Modified Si–O Interfaces in FeSiAl Soft Magnetic Composites

**DOI:** 10.3390/ma19204431

**Published:** 2026-10-21

**Authors:** Ziyi Luo, Yitong Liu, Yixin Liu, Haoze Chen, Qingchao Ding, Jiajian Chen, Jiantao Zhang, Zhiyu Xiao

**Affiliations:** 1 National Engineering Research Center of Near-Net-Shape Forming for Metallic Materials, South China University of Technology, Guangzhou 510640, China; lzyalyc@163.com (Z.L.);; 2 Guangdong Provincial Key Laboratory for Processing and Forming of Advanced Metallic Materials, South China University of Technology, Guangzhou 510640, China; 3 Guangdong YUEHAIHUAJIN Technology Co., Ltd., Guangzhou 511356, China; chenjj@hua-jin.com.cn; 4 Guangzhou City University of Technology, Guangzhou 510800, China

**Keywords:** FeSiAl soft magnetic composites, nano-oxide-modified Si–O interfaces, insulating interfaces, spatial distribution, local coordination, high-frequency magnetic properties

## Abstract

Existing strategies for modifying soft magnetic composites (SMCs) with nano-oxides primarily rely on their high electrical resistivity, with limited attention paid to interfacial coupling between the nano-oxides and the insulating matrix. This study proposes a high-temperature annealing strategy to regulate the spatial distribution and local coordination of nano-oxide-modified Si–O interfaces by incorporating Al_2_O_3_ and CeO_2_ into a silicone resin coating system, thereby harnessing their potential for interfacial modification at elevated temperatures. After annealing at 800 °C, the silicone resin forms an inorganic insulating interface characterized by an amorphous Si–O-rich network. The incorporation of Al_2_O_3_ and CeO_2_ alters the spatial distribution of the insulating interface and generates Si–O–Al/Al–O- and Si–O–Ce/Ce–O-related oxygen environments, respectively, thereby regulating the local coordination of the Si–O network. Al_2_O_3_ modification facilitates core-loss reduction, with the sample containing 0.5 wt.% Al_2_O_3_ achieving a core loss of 144.31 mW/cm^3^ at 100 kHz and 50 mT. In contrast, CeO_2_ incorporation is more favorable for maintaining high effective permeability, with the sample containing 1 wt.% CeO_2_ achieving a value of 97.88 at 100 kHz. These findings provide insights into the relationships among interfacial spatial distribution, local coordination, and high-frequency magnetic properties, offering guidance for the design of high-temperature composite insulating interfaces for FeSiAl SMCs.

## 1. Introduction

As power electronic devices evolve toward higher operating frequencies, smaller sizes, and greater power density, increasingly stringent requirements are being placed on the soft-magnetic properties of magnetic core materials [1,2,3,4]. FeSiAl soft magnetic composites (SMCs) offer favorable soft-magnetic properties, a low magnetostriction coefficient, and three-dimensional isotropic magnetic responses, making them promising candidates for high-frequency power electronic applications [5,6,7]. FeSiAl SMCs are typically fabricated through insulating coating, high-pressure compaction, and subsequent annealing, with their magnetic performance governed by both compaction-induced residual stress and interparticle insulation. High-temperature annealing facilitates residual-stress relaxation but also places greater demands on the thermal stability of the insulating layer [8,9]. Therefore, maintaining and further optimizing interparticle insulation while achieving sufficient stress relaxation represents an important approach to improving the magnetic properties of FeSiAl SMCs.

Currently, nanoscale inorganic particles, such as SiO_2_, TiO_2_, CaCO_3_, Fe_3_O_4_, Al_2_O_3_, and CeO_2_, are commonly incorporated into insulating layers as high-resistivity fillers to enhance interparticle electrical resistivity and suppress eddy-current loss [10,11,12,13,14]. Previous studies have primarily focused on the types and concentrations of these nanofillers and their effects on effective permeability and core loss, with particular emphasis on the intrinsic electrical insulating properties of inorganic nanoparticles. However, such filler-based modification strategies have paid relatively limited attention to interfacial coupling between the nanoparticles and the insulating matrix. Consequently, the potential of nano-oxides for insulating layer construction and interfacial chemical regulation remains insufficiently explored, which may limit further improvements in the magnetic performance of SMCs.

To address these issues, this study proposes a strategy for regulating the spatial distribution and local coordination of nano-oxide-modified Si–O interfaces through high-temperature annealing. Based on the high-temperature inorganic conversion of silicone resin, this study investigates the spatial distributions of Al_2_O_3_ and CeO_2_ within the composite insulating interface and their local chemical interactions with the silicone-resin-derived Si–O network. The aim is to explore the interfacial modification potential of nano-oxides under high-temperature treatment. On this basis, effective permeability, DC bias characteristics, and core loss were evaluated, while loss-separation analysis was employed to examine the relationship between interfacial structure and high-frequency energy dissipation. These findings provide a basis for optimizing the design of composite insulating interfaces in FeSiAl SMCs.

## 2. Experimental Methods

### 2.1. Materials

Gas-atomized spherical FeSiAl (Fe–9.6 wt.% Si–5.4 wt.% Al) powder with a median particle diameter (D50) of 29.83 μm was supplied by Guangdong Yuehaihuajin Co., Ltd., Guangzhou, China, Silicone resin (RSN-0805, Dow Corning, Midland, MI, USA) was used as the coating material, and xylene (Sinopharm Chemical Reagent Co., Ltd., Beijing, China) was used as the solvent. Al_2_O_3_ (99.9% purity) and CeO_2_ (99.5% purity) nanoparticles were supplied by Shanghai Macklin Biochemical Technology Co., Ltd., Shanghai, China.

### 2.2. Preparation of SMCs

**Figure 1:**
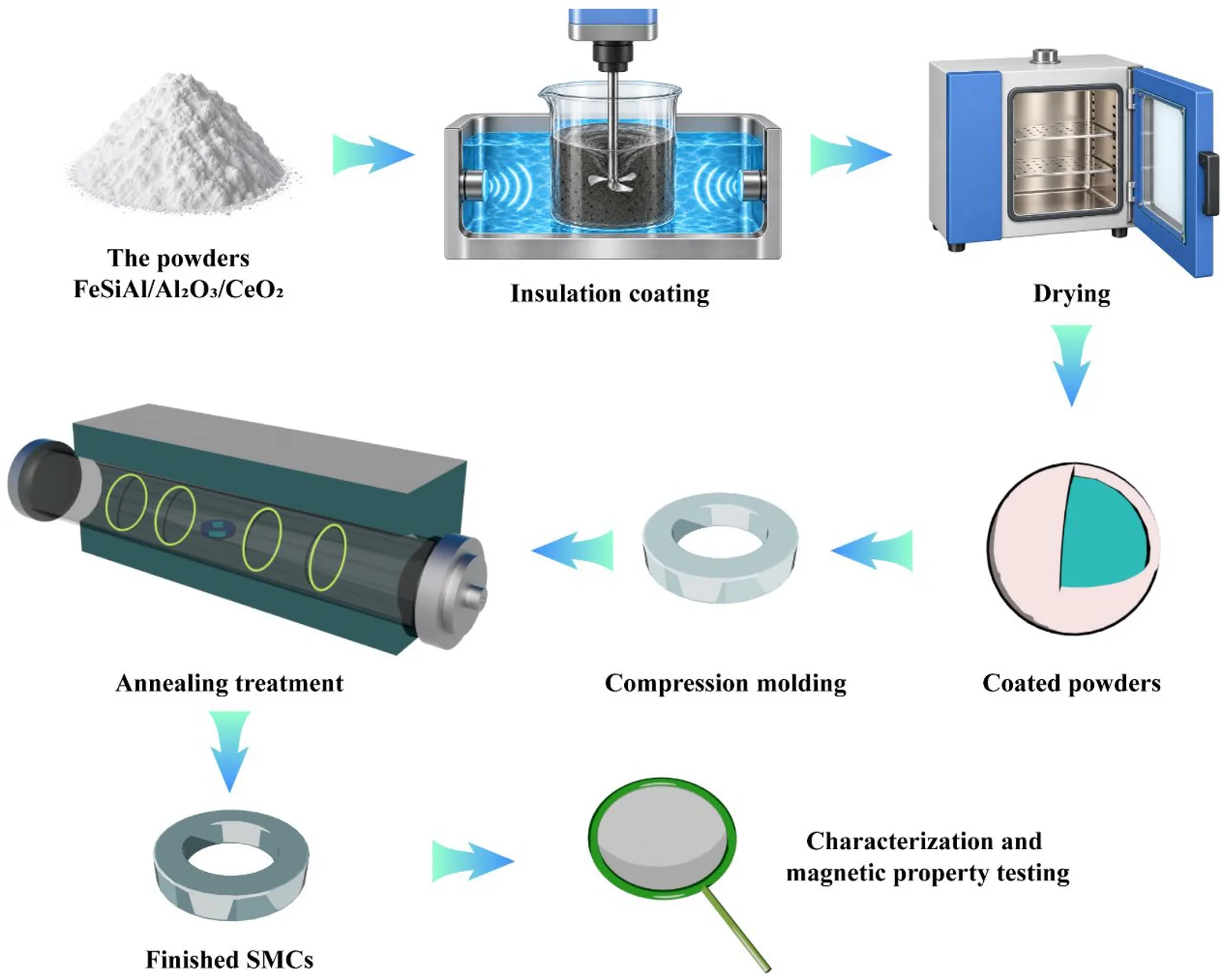
Preparation processes of the SMCs.

The preparation process of the SMCs is illustrated in Figure 1. FeSiAl powder, silicone resin (2 wt.%), and the corresponding nano-oxide additives were weighed according to the target compositions, with all mass fractions calculated relative to the mass of FeSiAl powder. For the unmodified samples, the silicone resin was dissolved in xylene and dispersed by ultrasonication-assisted mechanical stirring for 5 min. For the nano-oxide-modified samples, Al_2_O_3_ or CeO_2_ nanoparticles were first dispersed in xylene by ultrasonication for 5 min. Subsequently, the silicone resin was added, followed by ultrasonication-assisted mechanical stirring for another 5 min to obtain a composite coating solution. The ultrasonic frequency and power were 40 kHz and 120 W, respectively, and the mechanical stirring speed was 500 rpm.

The FeSiAl powder was then added to the coating solution and continuously mixed to promote uniform deposition of the insulating components on the particle surfaces. Stirring was maintained for approximately 4 h during solvent evaporation. The residual solvent was subsequently removed by natural evaporation, and the coated powder was dried at 120 °C for 1 h. Finally, the dried powder was gently ground and passed through a 60-mesh sieve to obtain the coated powder for subsequent compaction.

The coated powder was loaded into a toroidal die and compacted under a pressure of 1500 MPa to produce toroidal powder cores with an outer diameter of 16.5 mm, an inner diameter of 10.2 mm, and a height of 6.35 mm. The as-compacted cores were vacuum-annealed in a Kejing GSL-1400X tube furnace (Hefei Kejing Material Technology Co., Ltd., Hefei, China) at a heating rate of 3 °C min^−1^. After reaching the target temperature, the samples were held for 1 h and subsequently furnace-cooled to room temperature. The annealing temperatures ranged from 400 to 900 °C in increments of 100 °C, with the unannealed sample designated as Si2 and the annealed samples labeled Si2A400, Si2A500, Si2A600, Si2A700, Si2A800, and Si2A900, respectively; the nano-oxide contents were set at 0.25, 0.5, and 1 wt.%, with the Al_2_O_3_-modified samples denoted as A0.25, A0.5, and A1 and the CeO_2_-modified samples denoted as C0.25, C0.5, and C1, respectively. Pure silicone resin was designated as SR, while silicone resin modified with 0.5 wt.% Al_2_O_3_ and CeO_2_ was labeled SR-A and SR-C, respectively.

### 2.3. Materials Characterization

The microstructure and elemental distribution of the insulating coating were characterized by scanning electron microscopy (SEM, SU8220, Hitachi High-Technologies, Tokyo, Japan) equipped with an energy-dispersive X-ray spectroscopy (EDS) detector (X-MaxN, Oxford Instruments, Abingdon, UK). Phase identification was performed by X-ray diffraction (XRD, D8 DISCOVER, Bruker, Germany) using Cu Kα radiation over a 2θ range of 10–90°, with a step size of 0.0125° and a counting time of 0.2 s per step. The chemical bonding structure and surface chemical states of the coating were investigated by Fourier-transform infrared (FTIR) spectroscopy (Nicolet iN10, Thermo Fisher Scientific, Waltham, MA, USA) and X-ray photoelectron spectroscopy (XPS, ESCALAB 250Xi, Thermo Fisher Scientific, USA), respectively. The XPS binding energies were charge-corrected using the C–C/C–H component of the C 1s peak at 284.8 eV as the reference. The thermal behavior was investigated by simultaneous thermogravimetry and differential scanning calorimetry (TG-DSC, STA 449 F3, NETZSCH, Selb, Germany).

The effective permeability (μ_e_) of the magnetic cores was measured using an impedance analyzer (6632, MICROTEST, Taiwan, China) over a frequency range of 100 kHz to 1 MHz. The DC bias characteristics were evaluated under applied magnetic fields up to 100 Oe using an inductance DC bias test system (TH903A, Tonghui Electronics, Changzhou, China), and the permeability retention (%μ) was determined. The core loss (P_cv_) was measured using a soft magnetic material dynamic measurement system (MATS-3131SA, Linkjoin, Chengdu, China) over a frequency range of 50–300 kHz at a peak magnetic flux density (B_m_) of 50 mT. The density of the SMCs was determined using the Archimedes water-displacement method. The electrical resistivity of the SMCs was measured using a high-resistance microcurrent tester equipped with a toroidal three-electrode probe (ST2643-F02, Suzhou Lattice Electronics Co., Ltd., Suzhou China). At least three independently prepared powder cores were tested for each composition to assess the reproducibility of the magnetic measurements.

## 3. Results and Discussion

### 3.1. Thermal Conversion of Silicone Resin

**Figure 2:**
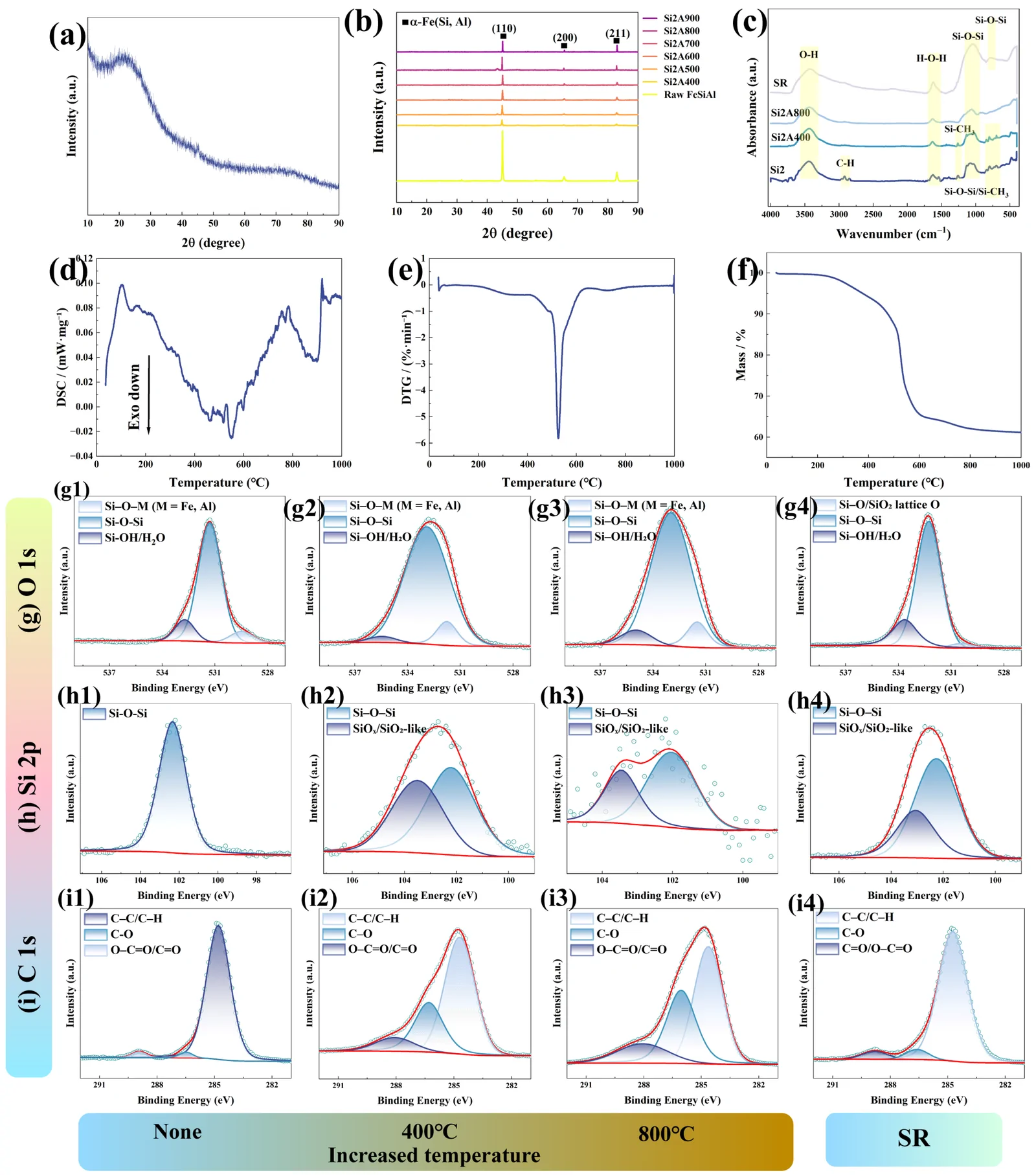
(**a**) XRD pattern of the SR residue after 800 °C annealing; (**b**) XRD patterns of raw FeSiAl powder and Si2 powders annealed at different temperatures; (**c**) FTIR spectra of Si2A400, Si2A800 and SR; (**d**) DSC, (**e**) DTG, and (**f**) TG curves of SR. High-resolution O 1s (**g1**–**g4**), Si 2p (**h1**–**h4**), and C 1s (**i1**–**i4**) XPS spectra and corresponding peak-fitting results for Si2 (**g1**,**h1**,**i1**), Si2A400 (**g2**,**h2**,**i2**), Si2A800 (**g3**,**h3**,**i3**), and SR (**g4**,**h4**,**i4**).

Figure 2 summarizes the thermal evolution of the silicone resin coating layer. Figure 2a presents the XRD pattern of the SR residue after annealing at 800 °C. A broad diffuse halo appears within 15–35°, whereas no distinct sharp diffraction peaks associated with crystalline phases are observed. This feature indicates that the heat-treated SR residue is predominantly amorphous, with no appreciable crystalline phases detectable by XRD. In addition, the XRD patterns in Figure 2b confirm that the FeSiAl matrix undergoes no evident transformation of its dominant phase over the temperature range of 400–900 °C, retaining the characteristic diffraction peaks of α-Fe(Si, Al).

Figure 2c traces the evolution of chemical bonding within the silicone resin coating layer as a function of annealing temperature. The absorption bands at approximately 3420–3445 cm^−1^ and 1618–1632 cm^−1^ are assigned to O–H stretching vibrations and H–O–H bending vibrations of adsorbed water, respectively. The band near 2916 cm^−1^ corresponds to C–H vibrations, while the absorption at 1260 cm^−1^ is associated with Si–CH_3_ groups [15,16]. The band at 797 cm^−1^ is attributed to symmetric Si–O–Si stretching and/or Si–CH_3_-related vibrations [17], while the broad band at 1000–1100 cm^−1^ is associated with Si–O–Si stretching vibrations [17]. After annealing at 400 °C, the C–H-related absorption disappears, although the characteristic Si–CH_3_ feature remains discernible. At 800 °C, the absorption bands associated with organic groups are markedly weakened, with no distinct C–H or Si–CH_3_ bands remaining, whereas the Si–O–Si absorption remains clearly identifiable. The SR residue annealed at 800 °C exhibits Si–O–Si characteristics similar to those of Si2A800. Collectively, these results demonstrate that progressive thermal treatment leads to the gradual decomposition and removal of organic side groups, while the Si–O–Si framework is retained at elevated temperatures.

The thermal analysis results presented in Figure 2d–f reveal distinct multistage mass-loss behavior of SR during heating. Below approximately 200 °C, the mass remains essentially unchanged. A gradual weight loss occurs between 200 and 450 °C, which is mainly associated with the removal of low-molecular-weight species and partial organic side groups. The principal thermal decomposition occurs between 450 and 600 °C, with the maximum mass-loss rate observed at approximately 520 °C, indicating extensive decomposition of the organic constituents. Above 600 °C, the rate of mass loss decreases markedly, and approximately 61% of the initial mass is retained even at 1000 °C, indicating that decomposition of the major organic constituents of the silicone resin leaves behind a thermally stable residual structure. This thermal behavior is consistent with the FTIR results, which document the progressive depletion of organic side groups together with the persistent presence of the Si–O–Si framework.

The high-resolution O 1s spectra in Figure 2(g1–g4) are dominated by Si–O–Si-related components at approximately 532 eV, accompanied by Si–OH/H_2_O species near 535 eV and lower-binding-energy components near 531 eV assigned to Si–O–M (M = Fe, Al) [18,19,20]. The latter may arise from Si–O–M-type interfacial environments involving the silicone-resin-derived Si–O network and Fe- or Al-containing surface oxides on the FeSiAl particles. As the annealing temperature increases, the Si–O–Si-associated oxygen environment persists throughout the investigated conditions, reflecting the favorable high-temperature stability of the Si–O–Si network.

The high-resolution Si 2p spectra contain a Si–O–Si component at approximately 102 eV for all samples [21]. Compared with the unannealed Si2 sample, Si2A400 and Si2A800 additionally exhibit a higher-binding-energy SiO_x_/SiO_2_-like Si–O component near 103 eV [19,22]. This evolution suggests that the decomposition of organic side groups is accompanied by changes toward more highly oxidized local Si–O environments. The SR residue annealed at 800 °C exhibits a comparable Si–O chemical environment, supporting that the Si–O structures detected in Si2A800 mainly originate from the high-temperature pyrolysis, condensation, and inorganic conversion of the silicone resin itself. Together with the amorphous XRD characteristics of the SR residue in Figure 2a, these Si–O-related features support the presence of an amorphous siloxane network with limited long-range structural order, rather than appreciable crystalline SiO_2_.

The C 1s spectra in Figure 2(i1–i4) exhibit components associated with C–C/C–H, C–O, and C=O/O–C=O environments. After annealing at 800 °C, carbon-related signals remain detectable in Si2A800 and the SR residue, which may originate from minor carbonaceous residues and adventitious surface carbon. Together with the FTIR and thermal analysis results, these observations indicate extensive decomposition of the original organic groups, although some carbon-containing species may remain in the high-temperature residue.

Taken together, the XRD, FTIR, TG–DTG–DSC, and XPS results indicate that the silicone resin coating undergoes a progressive transformation from an organosilicon structure containing organic side groups into a predominantly inorganic Si–O-rich network during high-temperature treatment. After annealing at 400 °C, organic moieties such as C–H and Si–CH_3_ are still retained within the coating layer. Within the principal decomposition range of 450–600 °C, extensive degradation of the organic constituents occurs, accompanied by structural rearrangement of the Si–O–Si network. Upon further heating to 800 °C, the signatures associated with organic side groups become markedly attenuated, whereas Si–O–Si species and more highly oxidized Si–O environments remain stable. Combined with the amorphous structural characteristics of the SR residue after annealing at 800 °C, these observations support the formation of a high-temperature insulating layer dominated by an amorphous Si–O-rich network. Although SiOC-related structures and free carbon have been reported in the pyrolysis products of polysiloxane-based precursors under oxygen-deficient conditions, their possible presence in the present residue cannot be conclusively determined from the available characterization results.

**Figure 3:**
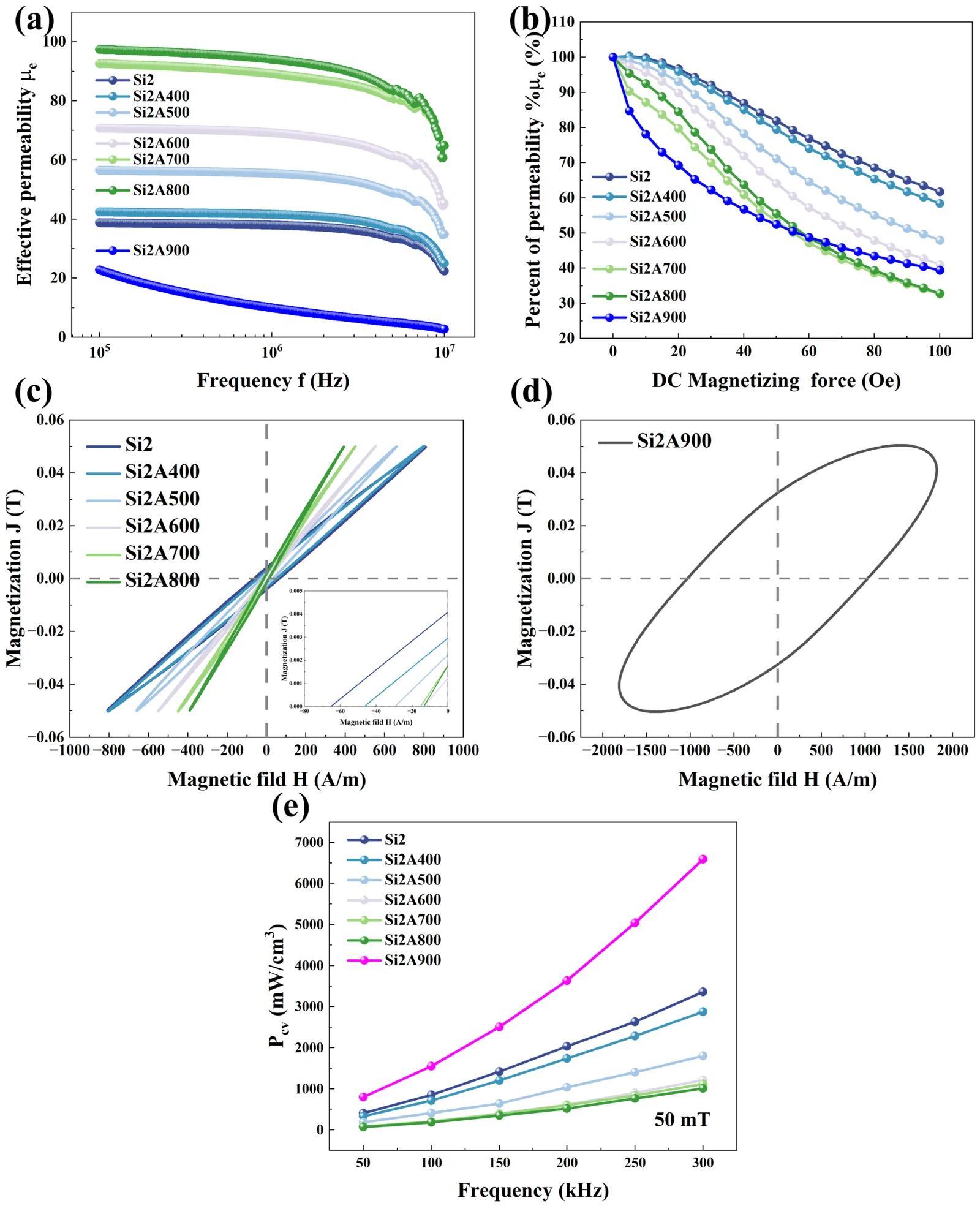
Magnetic properties of silicone-resin-coated FeSiAl SMCs before annealing and after annealing at different temperatures: (**a**) frequency dependence of effective permeability; (**b**) DC bias characteristics; (**c**) AC hysteresis loops of Si2 and Si2A400−Si2A800; (**d**) AC hysteresis loop of Si2A900; (**e**) frequency dependence of core loss at 50 mT.

The magnetic properties of Si2 and Si2A400–Si2A900 are summarized in Figure 3 and Table 1. Here, Si2 represents the FeSiAl sample coated with 2 wt.% silicone resin without annealing, while Si2A400–Si2A900 denote samples annealed at the corresponding temperatures of 400–900 °C, with “A” indicating annealing. As the annealing temperature increases to 800 °C, the effective permeability of the FeSiAl SMCs increases, with μ_e_ at 100 kHz rising from 38.74 for the unannealed Si2 to 97.39 for Si2A800. Concurrently, the core loss is substantially reduced; at 100 kHz and 50 mT, P_cv_ decreases from 850.29 to 183.53 mW/cm^3^. Further heating to 900 °C, however, leads to pronounced deterioration of the magnetic properties. For Si2A900, μ_e_ falls to 22.79, whereas P_cv_ reaches 1552.4 mW/cm^3^. Its AC hysteresis loop also becomes markedly broader and more rounded, indicating increased energy dissipation during dynamic magnetization and a pronounced degradation of the soft-magnetic response. Meanwhile, the electrical resistivity decreases sharply from 0.68 MΩ·cm for Si2A800 to 0.032 MΩ·cm for Si2A900, indicating a substantial deterioration in interparticle electrical insulation. Combined with the thermal analysis results, this suggests that excessive annealing may compromise the effectiveness of the silicone-resin-derived insulating interface, thereby contributing to the increased core loss.

The DC bias performance generally declines as μ_e_ becomes higher. At 100 Oe, the permeability retention ratio decreases progressively from 61.68% for Si2 to 32.55% and 32.74% for Si2A700 and Si2A800, respectively, reflecting the typical trade-off between effective permeability and DC bias stability. Although the permeability retention ratio of Si2A900 partially recovers, its initial μ_e_ has already undergone a substantial reduction; therefore, this change does not represent a genuine improvement in its DC bias performance.

The enhancement in magnetic performance within the range of 400–800 °C is mainly associated with the progressive release of residual stresses introduced during high-pressure compaction [8,23,24,25]. As the annealing temperature increases, residual stresses and their associated constraints on domain-wall motion are progressively reduced, facilitating magnetization and increasing μ_e_ while narrowing the AC hysteresis loops. Meanwhile, the structural characterization discussed above demonstrates that the silicone resin coating undergoes decomposition of organic side groups and reconstruction of the Si–O–Si network during high-temperature treatment, ultimately forming an inorganic insulating layer dominated by an amorphous Si–O-rich network after annealing at 800 °C. This high-temperature residual interface remains capable of maintaining electrical isolation between adjacent FeSiAl particles and suppressing the formation of interparticle conductive paths. Accordingly, the simultaneous improvement in effective permeability and reduction in core loss over 400–800 °C can be attributed to the combined effects of annealing-induced stress relaxation and preservation of the high-temperature insulating interface. At 900 °C, this balance is disrupted, as evidenced by the deterioration in interparticle insulation and dynamic magnetic response, resulting in a sharp decrease in effective permeability and a substantial increase in core loss.

### 3.2. Spatial Distribution of Nano-Al_2_O_3_/CeO_2_

**Figure 4:**
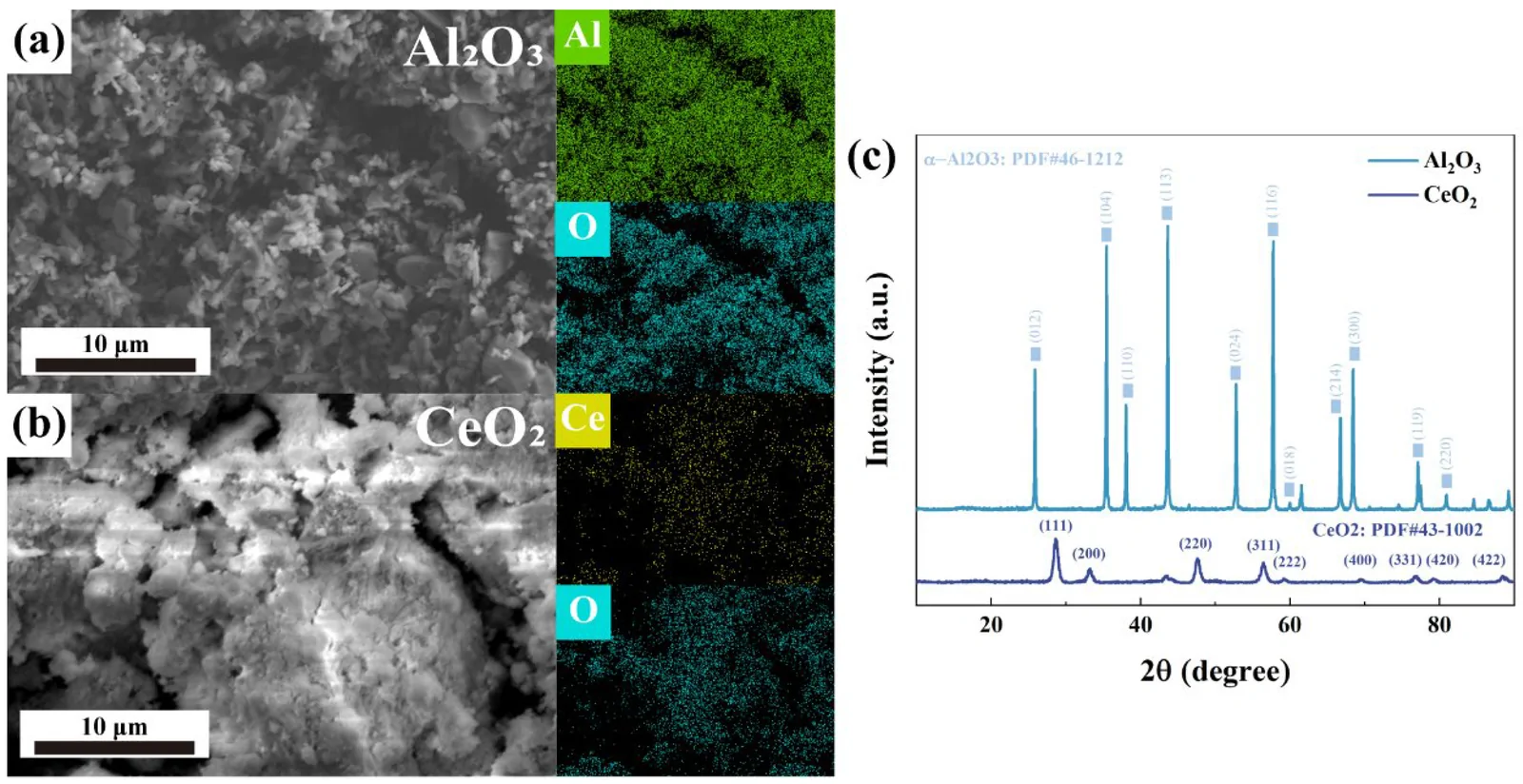
SEM images and corresponding EDS elemental maps of (**a**) Al_2_O_3_ and (**b**) CeO_2_ nanoparticles; (**c**) XRD patterns of Al_2_O_3_ and CeO_2_ nanoparticles.

Based on the thermal evolution of the silicone resin coating and the magnetic properties of the resulting SMCs, 800 °C was selected as the annealing temperature for the subsequent nano-oxide modification experiments. In the nano-oxide-modified series, A0.25, A0.5, and A1 represent samples containing 0.25, 0.5, and 1 wt.% Al_2_O_3_, respectively, while C0.25, C0.5, and C1 correspond to the same addition levels of CeO_2_. All samples in this series were annealed at 800 °C. Unlike the annealing-temperature series, “A” in these sample designations refers to Al_2_O_3_ rather than annealing. Figure 4a,b present the SEM morphologies and EDS elemental maps of the Al_2_O_3_ and CeO_2_ nanoparticles. According to the material specifications, the nominal particle sizes of Al_2_O_3_ and CeO_2_ are 30 nm and 20–50 nm, respectively. Owing to their high surface energy, both nanopowders exhibit secondary agglomeration in the as-received state. The Al_2_O_3_ nanopowder consists of fine particles assembled into micrometer-scale agglomerates with relatively loose and irregular morphologies. The corresponding EDS maps show a strong spatial correlation between Al and O in the particle-rich regions, consistent with the composition of Al_2_O_3_. In contrast, the CeO_2_ nanopowder exhibits more pronounced agglomeration, forming relatively dense, block-like aggregates. The EDS maps show that Ce and O are concentrated in these aggregates, consistent with the composition of CeO_2_. Overall, both nanopowders exhibit secondary agglomeration, with Al_2_O_3_ forming relatively loose clusters and CeO_2_ forming denser aggregates.

Figure 4c provides XRD patterns of the Al_2_O_3_ and CeO_2_ nanoparticles. The principal diffraction peaks of Al_2_O_3_ can be indexed to the (012), (104), (110), (113), (024), and (116) planes, consistent with the standard diffraction pattern of corundum-type α-Al_2_O_3_ (PDF No. 46-1212). For CeO_2_, the characteristic reflections corresponding to the (111), (200), (220), (311), and (222) planes agree well with the standard pattern of cubic fluorite CeO_2_ (PDF No. 43-1002). No discernible diffraction peaks associated with impurity phases are detected for either nanopowder.

**Figure 5:**
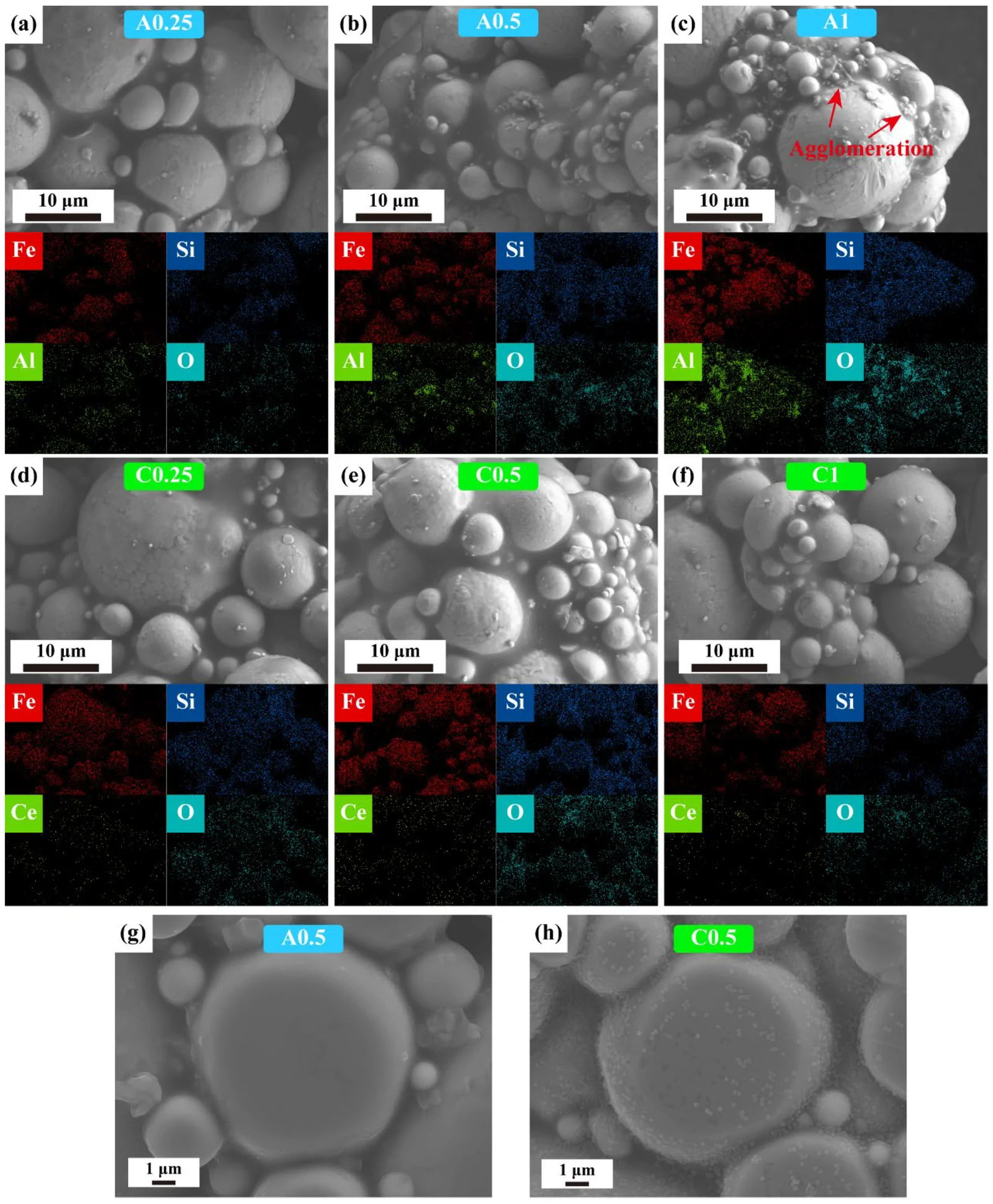
SEM images and corresponding EDS elemental maps of silicone-resin-coated FeSiAl powders modified with different Al_2_O_3_ and CeO_2_ contents: (**a**) A0.25, (**b**) A0.5, (**c**) A1, (**d**) C0.25, (**e**) C0.5, and (**f**) C1. Cross-sectional SEM images of (**g**) A0.5 and (**h**) C0.5 SMCs after annealing at 800 °C.

Figure 5a–f present SEM morphologies and EDS elemental maps of silicone-resin-coated FeSiAl powders modified with different amounts of Al_2_O_3_ and CeO_2_. In the Al_2_O_3_-modified series, only a small number of fine particles are observed on the FeSiAl particle surfaces in A0.25. At an Al_2_O_3_ content of 0.5 wt.%, the fine inorganic phase becomes more extensively distributed over the FeSiAl particle surfaces and within the interparticle regions. Raising the Al_2_O_3_ content to 1 wt.% gives rise to evident local particle enrichment and clustered structures on the surface of A1. The EDS maps reveal a good spatial correlation between Al and O over specific particle-surface regions. In A1, the agglomerated regions exhibit stronger Al and O signals, consistent with the particle clusters observed in the SEM images. By comparison, the inorganic phase in A0.5 is distributed more uniformly across the particle surfaces, without large-scale agglomeration.

A similar trend is observed in the CeO_2_-modified series, with increasing numbers of fine particles appearing on the FeSiAl surfaces as the CeO_2_ content increases. Only a few fine particles are observed on the FeSiAl surfaces in C0.25, whereas C0.5 exhibits a greater number of fine particles distributed over the particle surfaces and within the interparticle regions. At a CeO_2_ content of 1 wt.%, localized particle enrichment is observed in C1, although agglomeration is less pronounced than in A1. Because the FeSiAl matrix itself contains no Ce, the Ce signal in the EDS maps can serve as a relatively direct indicator of the spatial distribution of CeO_2_-related phases. Distinct Ce signals are detected in all three CeO_2_-modified samples, providing evidence for the incorporation of CeO_2_-related phases into the silicone resin coating system. These observations indicate that both Al_2_O_3_ and CeO_2_ are incorporated into the silicone-resin-based composite coating, with their addition levels influencing the spatial distribution of the inorganic particles on the FeSiAl surfaces. At low loadings, the inorganic phase provides limited surface coverage, whereas excessive addition tends to favor local enrichment and agglomeration.

Figure 5g,h further compare the cross-sectional microstructures of A0.5 and C0.5 SMCs after annealing at 800 °C. In both samples, the outlines of the FeSiAl particles remain clearly distinguishable, and well-defined interfacial regions can still be identified between adjacent particles. No obvious large-scale sintering or complete fusion between adjacent particles is observed, suggesting that the interparticle boundaries are largely preserved after annealing at 800 °C. Notably, the two nano-oxide-modified systems display different spatial distributions at the particle interfaces following heat treatment. In C0.5, numerous fine particles are distributed relatively densely and continuously along the FeSiAl particle surfaces and interparticle boundaries, forming a distinct particulate interfacial structure. In contrast, A0.5 exhibits a more dispersed interfacial particle distribution, without the dense particulate features observed in C0.5. These morphological differences suggest that, under identical additive contents and annealing conditions, Al_2_O_3_ and CeO_2_ adopt distinct spatial distributions at the FeSiAl particle interfaces, which may further influence the interfacial continuity of the composite insulating layer and the degree of electrical isolation between neighboring particles.

### 3.3. Local Coordination of Nano-Al_2_O_3_/CeO_2_

**Figure 6:**
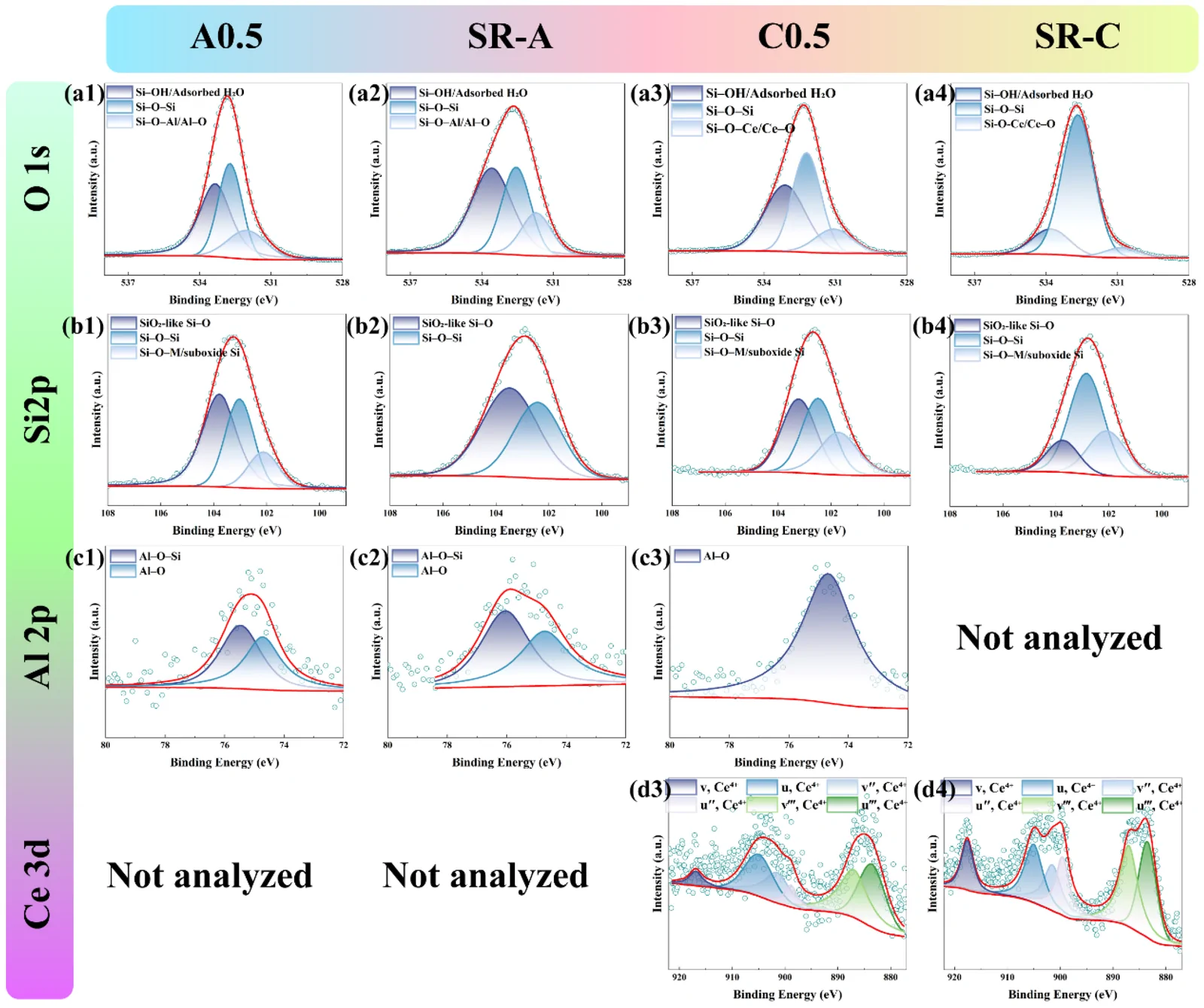
High-resolution XPS spectra and peak-fitting results of Al_2_O_3_/CeO_2_−modified silicone-resin-coated FeSiAl powders and the corresponding SR residues after annealing at 800 °C: O 1s (**a1**–**a4**), Si 2p (**b1**–**b4**), Al 2p (**c1**–**c3**), and Ce 3d (**d3**–**d4**).

The high-resolution XPS spectra of A0.5, C0.5, and the corresponding SR-A and SR-C samples are presented in Figure 6. The O 1s spectra provide information on the local oxygen chemical environments after the introduction of nano-oxides. For A0.5 and SR-A, the O 1s envelopes can be deconvoluted into three components located at 533.3–533.6 eV, 532.6–532.7 eV, and 531.7–532.0 eV, which are assigned to Si–OH/adsorbed H_2_O, Si–O–Si, and Si–O–Al/Al–O-related species, respectively. The latter component accounts for approximately 17.6% and 17.7% of the total O 1s area in A0.5 and SR-A. For the CeO_2_-modified system, the corresponding components in C0.5 occur at 533.12, 532.21, and 531.06 eV, representing Si–OH/adsorbed H_2_O, Si–O–Si, and Ce–O/Si–O–Ce-related oxygen species, respectively. In SR-C, the three fitted contributions are centered at 533.81, 532.66, and 530.81 eV. Among them, the Si–O–Si component at 532.66 eV is predominant, whereas the lower-binding-energy contribution at 530.81 eV is assigned to Ce–O/Si–O–Ce-related oxygen species. The O 1s fitting results suggest that distinct Si–O–Si network characteristics are retained in all samples after annealing at 800 °C, while the incorporation of Al_2_O_3_ or CeO_2_ modifies the local coordination environment of oxygen.

The high-resolution Si 2p spectra of all four samples are dominated by Si–O-related components associated with different local coordination environments. In A0.5, three components are located at 103.79, 103.03, and 102.12 eV and can be assigned to SiO_2_-like Si–O, Si–O–Si, and lower-binding-energy Si–O–M/suboxide Si-related environments, respectively. SR-A, by comparison, is mainly composed of two Si–O contributions at 103.48 and 102.41 eV. For C0.5, the three peaks are centered at 103.22, 102.49, and 101.71 eV, whereas the corresponding positions in SR-C are 103.74, 102.84, and 102.07 eV. Previous XPS studies on SiO_x_ systems have reported that, relative to fully oxidized Si^4+^, the Si 2p signals associated with intermediate Si^1+^–Si^3+^ oxidation states (suboxide Si) generally shift toward lower binding energies [26,27,28,29]. Accordingly, the component near 101.7–102.1 eV may reflect a lower-oxygen-coordination Si–O environment with incomplete SiO_2_-type coordination. Meanwhile, the present system also contains Al_2_O_3_, CeO_2_, and FeSiAl particle interfaces. Participation of Al, Ce, and other interfacial species in local Si–O coordination may generate Si–O–M environments and alter the local charge distribution around Si, thereby contributing to a shift of Si 2p toward lower binding energy. When considered together with the Al/Ce-related interfacial oxygen components in O 1s and the Al 2p and Ce 3d spectra, the low-binding-energy contribution can therefore be assigned to a Si–O–M/suboxide Si-related chemical environment. This component reflects changes in the local coordination environment of Si following nano-oxide incorporation and cannot be uniquely assigned to a specific Si suboxide species.

The high-resolution Al 2p spectra indicate that Al predominantly remains in an oxidized state in the Al_2_O_3_-modified system. SR-A, representing the Al_2_O_3_-modified silicone-resin residue after annealing at 800 °C without the FeSiAl substrate, was used as a reference to distinguish the contributions of the added Al_2_O_3_ and the FeSiAl matrix. The Al 2p envelope of A0.5 can be resolved into two components at 74.72 and 75.46 eV, while the corresponding peaks in SR-A are located at 74.73 and 76.05 eV. The component near 74.7 eV is associated with Al–O, corresponding to an Al_2_O_3_-like Al^3+^–O environment, whereas the higher-binding-energy contribution is related to Al–O–Si-type local coordination [30,31,32]. The nearly identical Al–O binding energies of A0.5 and SR-A suggest similar local chemical environments, while the 0.59 eV difference in their Al–O–Si-related components may reflect variations in local coordination and the influence of the FeSiAl substrate [33,34]. The high-binding-energy component accounts for approximately 53.2% and 53.6% of the total Al 2p area in A0.5 and SR-A, respectively. In conjunction with the O 1s and Si 2p results, these features suggest that Al_2_O_3_ largely retains its oxide-like chemical state after high-temperature treatment, while appreciable interfacial coupling develops with the silicone-resin-derived Si–O network, leaving part of the Al in an Al–O–Si-type local coordination environment [30,31,32]. As no additional Al_2_O_3_ is introduced into C0.5, its Al 2p spectrum contains only an Al–O component near 74.67 eV, originating mainly from oxidized Al species on the FeSiAl matrix surface. Therefore, although the comparison with SR-A supports the contribution of nano-Al_2_O_3_ to the Al-related environments in A0.5, the contribution from surface-oxidized Al in the FeSiAl substrate cannot be completely excluded.

Both C0.5 and SR-C exhibit the characteristic multiplet features of Ce^4+^. For C0.5, the v, v″, and v‴ components on the Ce 3d_5/2_ side are located at 883.55, 887.00, and 898.87 eV, respectively, while the corresponding u, u″, and u‴ components on the Ce 3d_3/2_ side occur at 901.55, 905.00, and 916.87 eV. The respective peak positions in SR-C are 883.48, 887.03, 899.58, 901.49, 905.03, and 917.58 eV [35]. The u‴ feature at approximately 916.9–917.6 eV is a characteristic signature of the Ce^4+^/CeO_2_-type chemical state [36], supporting that Ce remains predominantly in the Ce^4+^ oxidation state in both samples after annealing at 800 °C. Compared with SR-C, C0.5 exhibits a lower proportion of the high-binding-energy v‴/u‴ components, suggesting that the Si–O network and interfacial environment in the FeSiAl coating system may influence the local electronic structure of Ce. Given the low Ce content in C0.5 and the limited signal-to-noise ratio of the spectra, the Ce^3+^/Ce^4+^ ratio is not further quantified in this work.

Compared with Si2A800 without nano-oxide addition in Section 3.1, both A0.5 and C0.5 retain distinct Si–O–Si features and highly oxygen-coordinated Si–O environments after annealing at 800 °C, suggesting that the incorporation of Al_2_O_3_ or CeO_2_ does not alter the fundamental evolution pathway toward an inorganic insulating layer dominated by an amorphous Si–O-rich network. The modified systems differ, however, in the emergence of Al/Ce-associated interfacial oxygen environments together with lower-binding-energy Si–O-related components. These characteristics imply that the principal role of the nano-oxides is to regulate the local coordination of the silicone-resin-derived Si–O network and its interfacial chemical environment.

**Figure 7:**
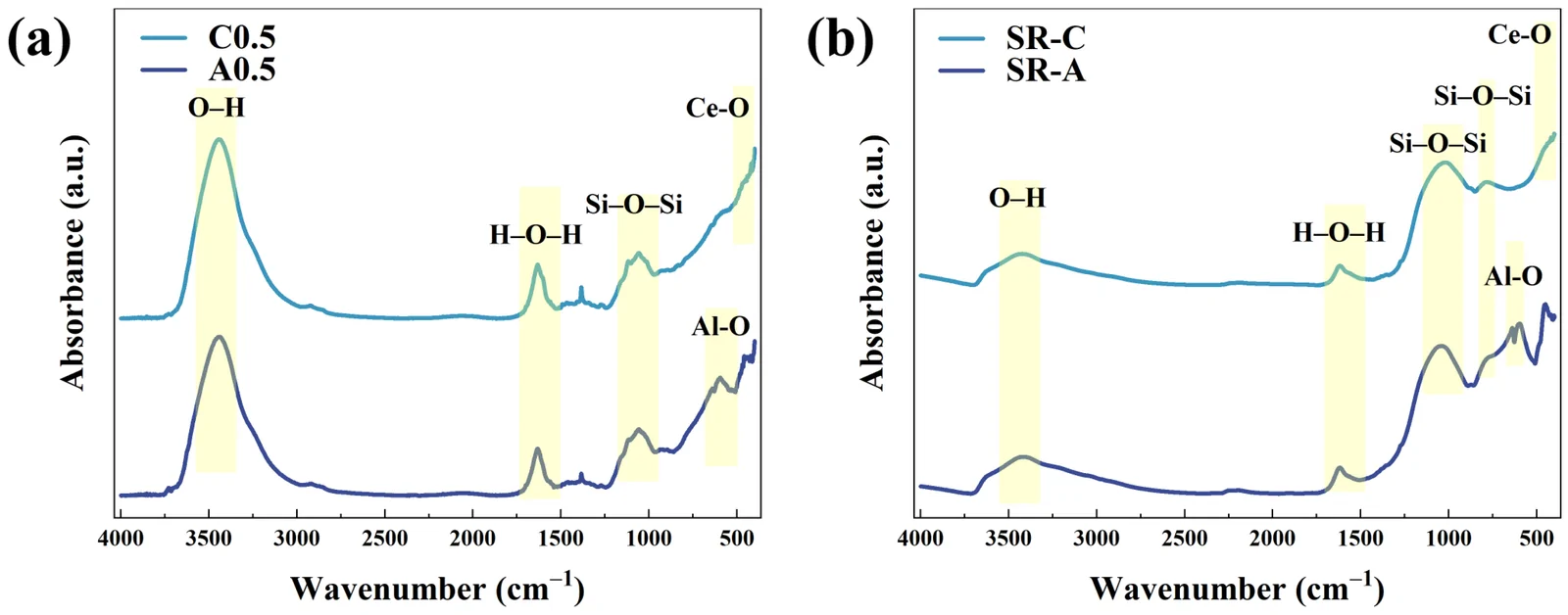
FTIR spectra of Al_2_O_3_/CeO_2_-modified silicone−resin−coated FeSiAl powders and the corresponding SR residues after annealing at 800 °C: (**a**) A0.5 and C0.5; (**b**) SR−A and SR−C.

The FTIR spectra of A0.5 and C0.5 powders annealed at 800 °C, together with those of the corresponding silicone resin residues (SR-A and SR-C), are presented in Figure 7. All four samples exhibit absorption bands at approximately 3420–3445 cm^−1^ and 1618–1632 cm^−1^, which are mainly assigned to O–H stretching vibrations and H–O–H bending vibrations of adsorbed water, respectively. A distinct Si–O–Si stretching band is observed near 1057 cm^−1^ in every sample, suggesting that, after the incorporation of Al_2_O_3_ or CeO_2_, the insulating residue produced by high-temperature conversion of the silicone resin at 800 °C remains dominated by a Si–O network. In contrast to Si2A800, additional features appear in the low-wavenumber region. A0.5 and SR-A display an absorption response near 600 cm^−1^ associated with Al–O lattice vibrations, while SR-A also contains low-frequency bands around 640 and 450 cm^−1^. For C0.5 and SR-C, a pronounced response emerges near 419 cm^−1^ and can be related to Ce–O lattice vibrations. Because this spectral region may also contain contributions from Si–O–Si bending modes and vibrations of other inorganic oxides, these assignments are regarded primarily as supplementary evidence for the presence of Al_2_O_3_ and CeO_2_ within the high-temperature residual insulating layer.

The principal Si–O–Si absorption features of SR−A and SR−C are similar to those of their corresponding A0.5 and C0.5 samples. This similarity suggests that, even in the absence of the FeSiAl matrix, Al_2_O_3_- or CeO_2_-modified silicone resin still converts at 800 °C into an inorganic residue dominated by a siloxane network. The incorporation of the nano-oxides therefore does not appear to alter the fundamental high-temperature inorganic conversion pathway of the silicone resin toward a Si–O-rich structure, but instead mainly contributes stable inorganic phases that regulate the composition of the composite insulating layer.

### 3.4. Magnetic Properties

**Figure 8:**
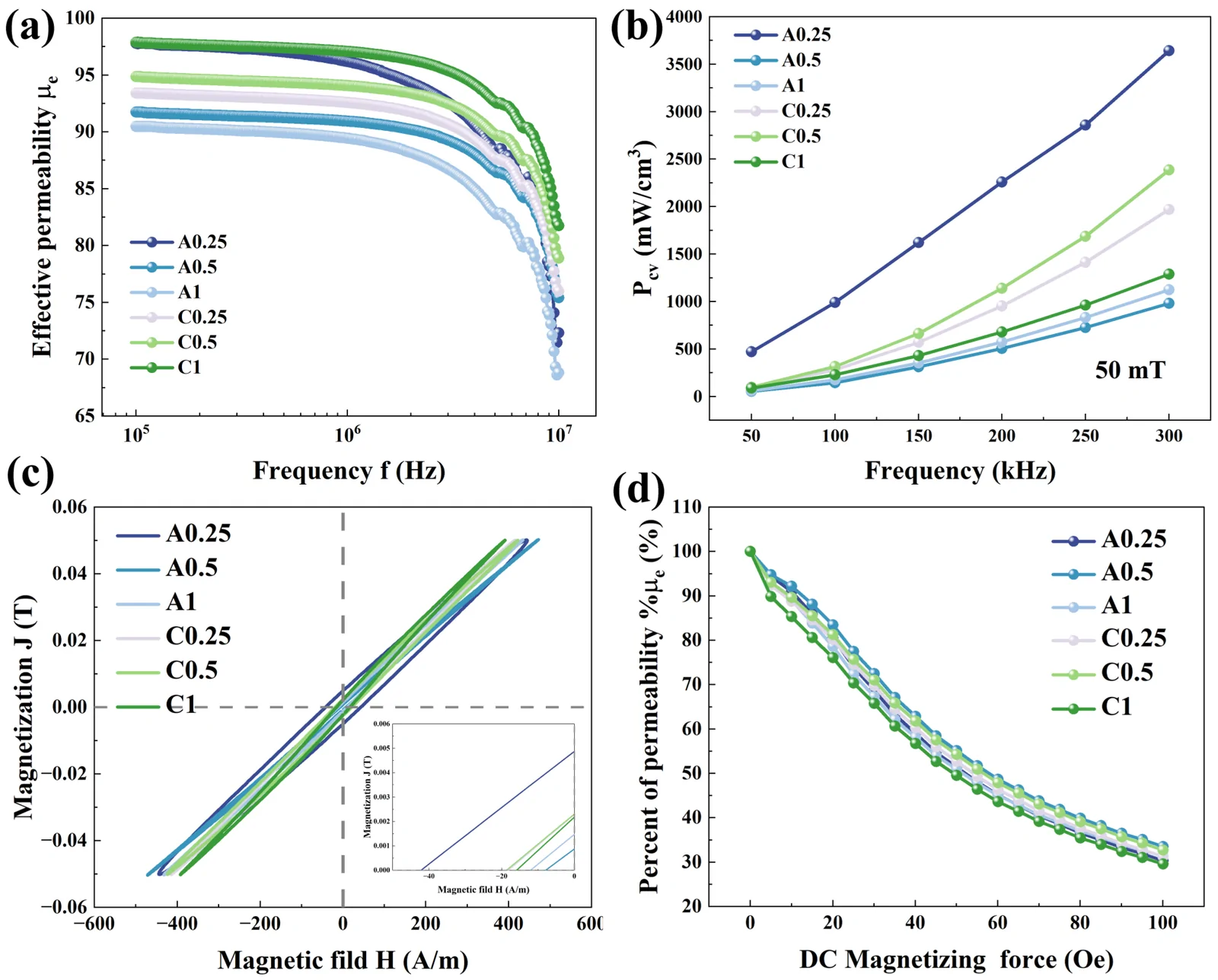
Magnetic properties of silicone-resin-coated FeSiAl SMCs modified with different contents of Al_2_O_3_ and CeO_2_ after annealing at 800 °C: (**a**) frequency dependence of effective permeability; (**b**) frequency dependence of core loss at 50 mT; (**c**) AC hysteresis loops; and (**d**) DC bias characteristics.

The magnetic properties of the powder cores containing different types and amounts of nano-oxides are summarized in Figure 8 and Table 2. All samples exhibit relatively stable effective permeability over the low- and intermediate-frequency ranges, followed by a gradual decline at higher frequencies. At 100 kHz, the Al_2_O_3_-modified series exhibits an overall downward tendency in μ_e_ with greater additive content, decreasing from 97.79 for A0.25 to 91.74 and 90.48 for A0.5 and A1, respectively. By contrast, the CeO_2_-containing samples follow a different dependence on composition. The μ_e_ values of C0.25, C0.5, and C1 at 100 kHz are 93.41, 94.87, and 97.88, respectively, corresponding to a progressive enhancement with increasing CeO_2_ loading.

More pronounced differences in core loss are observed among the modified systems. Within the Al_2_O_3_ series, A0.25 exhibits comparatively high loss throughout the entire measured frequency range. At an Al_2_O_3_ content of 0.5 wt.%, the P_cv_ of A0.5 is markedly reduced and reaches 144.31 mW/cm^3^ at 100 kHz and 50 mT, representing the lowest value among all modified samples. Raising the Al_2_O_3_ fraction further to 1 wt.% causes P_cv_ to rebound in A1. The CeO_2_ series displays a non-monotonic dependence of loss on additive level. At 100 kHz and 50 mT, the P_cv_ values of C0.25, C0.5, and C1 are 281.57, 315.75, and 228.71 mW/cm^3^, respectively, with C1 giving the minimum loss within the CeO_2_-modified group. Particularly at the same addition level of 0.5 wt.%, a substantial difference is observed between A0.5 and C0.5: their P_cv_ values are 144.31 and 315.75 mW/cm^3^, respectively, corresponding to a reduction of approximately 54.3% for A0.5.

The AC hysteresis loops in Figure 8c reveal distinct differences in the dynamic magnetization behavior of the nano-oxide-modified samples. Notably, A0.25 exhibits the largest hysteresis loop area, indicating enhanced energy dissipation during cyclic magnetization, consistent with its unusually high core loss. The other samples exhibit relatively narrow hysteresis loops, reflecting lower energy dissipation during cyclic magnetization. Figure 8d compares their DC bias performance. At 100 Oe, the permeability retention ratios of A0.25, A0.5, and A1 are 30.33%, 33.52%, and 31.29%, respectively, with A0.5 giving the highest value. The corresponding values for C0.25, C0.5, and C1 are 31.25%, 32.67%, and 29.60%, and the maximum retention ratio in this series is likewise obtained at 0.5 wt.% addition. C1 possesses the highest effective permeability of 97.88 at 100 kHz, while its permeability retention ratio at 100 Oe is the lowest, at 29.60%, reflecting the typical trade-off between effective permeability and DC bias retention.

The pronounced increase in core loss observed for A0.25 may be associated with the unfavorable spatial distribution of Al_2_O_3_ nanoparticles at a low loading of 0.25 wt.%. As shown in Figure 5a, only a limited number of fine inorganic particles are attached to the FeSiAl particle surfaces, indicating insufficient surface coverage by Al_2_O_3_. After annealing at 800 °C, such insufficient spatial coverage may result in a heterogeneous composite insulating interface, where Si–O–Al-related interfacial environments form in some regions containing Al_2_O_3_, while other regions remain dominated by the silicone-resin-derived Si–O network. Compared with the unmodified Si2A800 sample, the introduction of a small amount of Al_2_O_3_ may locally disturb the original coating structure without establishing a sufficiently uniform composite insulating interface. This interfacial heterogeneity could affect the uniformity and continuity of interparticle insulation, increasing the possibility of local electrical contact between adjacent FeSiAl particles and associated eddy-current losses [24]. Moreover, variations in the local interfacial structure and particle contact conditions may influence magnetization reversal and enhance energy dissipation associated with irreversible magnetization processes. When the Al_2_O_3_ content increases to 0.5 wt.%, the inorganic phase becomes more uniformly distributed over the FeSiAl particle surfaces, as observed in Figure 5b. Such improved spatial uniformity may help maintain interparticle electrical isolation and reduce local variations in magnetization behavior, thereby contributing to the substantially lower core loss of A0.5 [37].

The measured densities of Si2A800, A0.5, and C0.5 were 6.08, 5.92, and 6.03 g/cm^3^, respectively, with corresponding electrical resistivities of 0.68, 0.79, and 0.61 MΩ·cm. Compared with Si2A800, A0.5 exhibits a higher electrical resistivity and lower core loss, suggesting that the relatively uniform distribution of Al_2_O_3_-containing phases may contribute to improved interparticle electrical insulation. In contrast, C0.5 exhibits a lower resistivity and higher core loss despite the more continuous particulate interfacial distribution observed in SEM images. This suggests that the morphological continuity of the nano-oxide-containing phase does not necessarily correspond to improved electrical insulation. Furthermore, the higher density of C0.5 compared with A0.5 is consistent with its higher effective permeability, indicating that differences in particle packing and interparticle nonmagnetic spacing may also contribute to the magnetic response.

In conjunction with the preceding microstructural and interfacial chemical analyses, the influence of nano-oxides on the magnetic properties of FeSiAl SMCs can be considered from two interrelated aspects: interfacial spatial distribution and local coordination. The dispersion, local enrichment, and agglomeration of nano-oxides on the particle surfaces can modify the interparticle nonmagnetic spacing, local magnetic reluctance, and continuity of electrical insulation. The relatively uniform and continuous interfacial distribution of CeO_2_ helps avoid large local nonmagnetic gaps, thereby helping to preserve magnetic-path continuity. In the Al_2_O_3_-modified system, an appropriate and homogeneous distribution of the inorganic phase is more favorable for establishing effective electrical isolation between neighboring particles and restricting high-frequency conductive paths, which is consistent with its more pronounced suppression of core loss. In addition, the introduction of Al_2_O_3_ and CeO_2_ produces distinct Al- and Ce-involved interfacial oxygen environments and correspondingly modifies the local coordination of the Si–O network. The Al_2_O_3_-modified system is mainly characterized by Si–O–Al/Al–O-related interfacial oxygen environments, whereas the CeO_2_-containing system is associated with Si–O–Ce/Ce–O-related environments. Both systems are accompanied by variations in the lower-binding-energy Si–O components, suggesting different modes of regulating the local chemical state of the Si–O network. Taken together, the evolution of magnetic performance may arise from the combined effects of nano-oxide spatial distribution at the interfaces and the resulting local coordination environments.

According to the classical loss separation theory, the total core loss of soft magnetic materials can be expressed as the sum of hysteresis loss (P_h_), eddy-current loss (P_e_), and excess loss (P_ex_) [38,39]:

(1) $$\begin{array}{c} P_{\mathrm{cv}}=P_{h}+P_{e}+P_{\mathrm{ex}} \end{array}$$

Under a constant peak magnetic flux density, the frequency dependence of each loss component can be written as:

(2) $$\begin{array}{c} P_{h}=K_{h}f \end{array}$$

(3) $$\begin{array}{c} P_{e}=K_{e}f^{2} \end{array}$$

(4) $$\begin{array}{c} P_{\mathrm{ex}}=K_{\mathrm{ex}}f^{1.5} \end{array}$$

Accordingly, the total core loss is given by:

(5) $$\begin{array}{c} P_{\mathrm{cv}}=K_{h}f+K_{e}f^{2}+K_{\mathrm{ex}}f^{1.5} \end{array}$$

where f is the operating frequency, and K_h_, K_e_, and K_ex_ are the fitting coefficients associated with hysteresis loss, eddy-current loss, and excess loss, respectively. In the present loss-separation analysis, the peak magnetic flux density was fixed at B_m_ = 50 mT; therefore, the magnetic-flux-density-dependent terms are incorporated into the corresponding fitting coefficients.

The fitting coefficients K_h_, K_e_, and K_ex_ were determined by applying non-negative least-squares (NNLS) fitting directly to the experimentally measured total core losses over the frequency range of 50–300 kHz. The coefficients were constrained to be non-negative to avoid physically meaningless negative loss contributions. The frequency f was expressed in kHz, and the resulting coefficients were used to calculate the individual loss components at each measured frequency:

(6) $$\begin{array}{c} K_{h}\geq 0,K_{e}\geq 0,\;\;\;\;\;K_{\mathrm{ex}}\geq 0 \end{array}$$

Since the frequency f was expressed in kHz, the numerical values of K_h_, K_e_, and K_ex_ depend on the frequency unit adopted. These coefficients were therefore used to calculate the loss components of each sample rather than being compared as independent material constants. Once the fitting coefficients were obtained, P_h_, P_e_, and P_ex_ were calculated at each test frequency, and their sum was used to reconstruct the corresponding total core loss. To evaluate the relative contribution of each component at different frequencies, the percentage of each loss term in the total fitted loss was further calculated as:

(7) $$\begin{array}{c} \eta_{h}=\frac{P_{h}}{P_{h}+P_{e}+P_{\mathrm{ex}}}\times 100\% \end{array}$$

(8) $$\begin{array}{c} \eta_{e}=\frac{P_{e}}{P_{h}+P_{e}+P_{\mathrm{ex}}}\times 100\% \end{array}$$

(9) $$\begin{array}{c} \eta_{\mathrm{ex}}=\frac{P_{\mathrm{ex}}}{P_{h}+P_{e}+P_{\mathrm{ex}}}\times 100\% \end{array}$$

where η_h_, η_e_, and η_ex_ denote the percentages of hysteresis loss, eddy-current loss, and excess loss in the total fitted loss, respectively. The relative contributions of P_h_, P_e_, and P_ex_ were determined using the procedure described above. It should be noted that when a fitting coefficient approaches zero under the non-negative constraint, the corresponding loss term makes only a limited independent contribution within the investigated frequency range and the adopted fitting model; this does not imply the complete absence of the associated physical loss mechanism. Therefore, the loss-separation results are interpreted in conjunction with the total core loss and microstructural evolution.

The goodness of fit was evaluated using the coefficient of determination (R^2^) and root mean square error (RMSE) by comparing the experimentally measured total core losses with those reconstructed from the fitted loss components. The R^2^ values for Si2A800, A0.5, and C0.5 were 0.999582, 0.999557, and 0.999907, respectively, with corresponding RMSE values of 6.661, 6.828, and 7.669 mW/cm^3^. These results indicate that the adopted model provides a good reconstruction of the total core loss over the investigated frequency range. Nevertheless, a good fit to the total loss does not necessarily ensure the unique determination of the individual loss components. Independent estimation of hysteresis loss would require quasi-static or sufficiently low-frequency hysteresis measurements, which were not performed in the present study. Therefore, the fitted loss components are interpreted as model-dependent estimates of the frequency-dependent loss contributions rather than independently validated absolute values.

**Figure 9:**
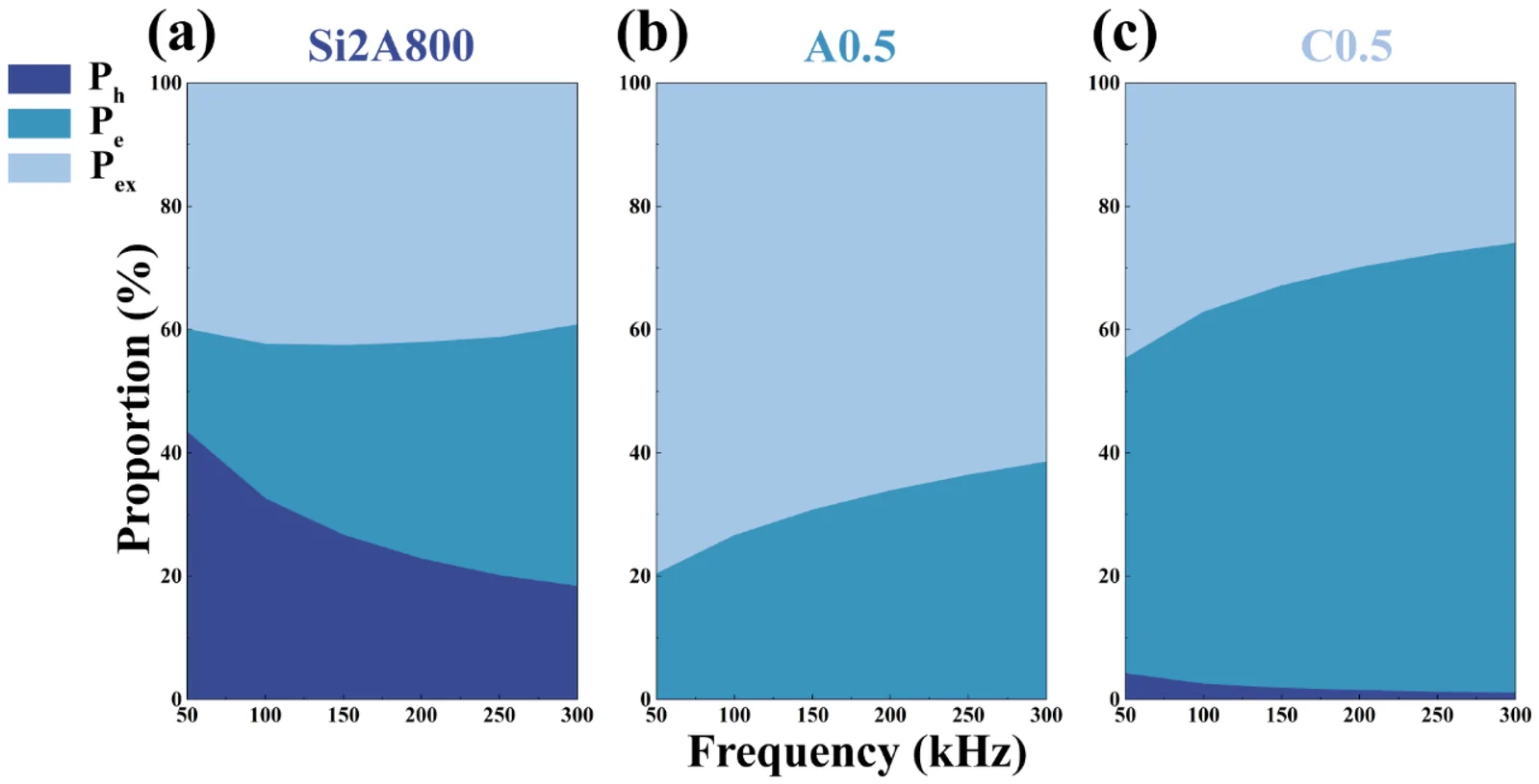
Frequency dependence of the fitted proportions of hysteresis loss (P_h_), eddy-current loss (P_e_), and excess loss (P_ex_) in (**a**) Si2A800, (**b**) A0.5, and (**c**) C0.5 SMCs at a peak magnetic flux density of 50 mT.

Figure 9 compares the relative contributions of P_h_, P_e_, and P_ex_ for Si2A800, A0.5, and C0.5 over the frequency range of 50–300 kHz at 50 mT. For Si2A800, the three loss components undergo a relatively continuous redistribution with frequency. At 300 kHz, P_h_, P_e_, and P_ex_ account for 18.32%, 42.41%, and 39.27% of the total fitted loss, respectively, suggesting that no single loss channel remains overwhelmingly dominant across the investigated frequency range for the baseline Si–O–Si interface after high-temperature inorganic conversion. For A0.5, the non-negative fitting procedure yields a near-zero hysteresis-loss coefficient, indicating that the hysteresis contribution cannot be independently resolved within the adopted model and frequency range, rather than implying the physical absence of hysteresis loss. Accordingly, the fitted loss is primarily represented by the excess- and eddy-current-loss terms, although their individual contributions should be interpreted with caution. Specifically, the fitted fraction of P_ex_ decreases from 79.7% to 61.5%, whereas that of P_e_ increases from 20.3% to 38.5%, suggesting a substantial contribution from loss processes associated with dynamic nonuniform magnetization within the adopted model. By contrast, the fitted P_e_ contribution in C0.5 increases from approximately 51.2% to 73.0% and becomes the predominant loss component within the adopted model, suggesting an increasingly prominent eddy-current-related contribution at higher frequencies. Although C0.5 exhibits a relatively continuous particulate interfacial distribution in the SEM images, such morphological continuity does not necessarily indicate more effective electrical insulation. The measured electrical resistivity of C0.5 (0.61 MΩ·cm) is lower than that of A0.5 (0.79 MΩ·cm), suggesting that the Al_2_O_3_-modified interface provides more effective overall electrical isolation. This difference is consistent with the higher fitted eddy-current-loss contribution of C0.5. Therefore, the spatial continuity of the oxide-containing interfacial structure should be distinguished from its electrical insulating effectiveness. These results suggest that the introduction of Al_2_O_3_ and CeO_2_ modifies not only the overall core-loss level but also the relative weighting of the underlying loss mechanisms. A0.5 is characterized predominantly by P_ex_-related dynamic magnetization loss, whereas C0.5 exhibits a distinct P_e_-dominated response, indicating that the composite interfaces produced by different nano-oxide modifications influence high-frequency dynamic magnetization and current-related dissipation through different pathways.

## 4. Conclusions

**Figure 10:**
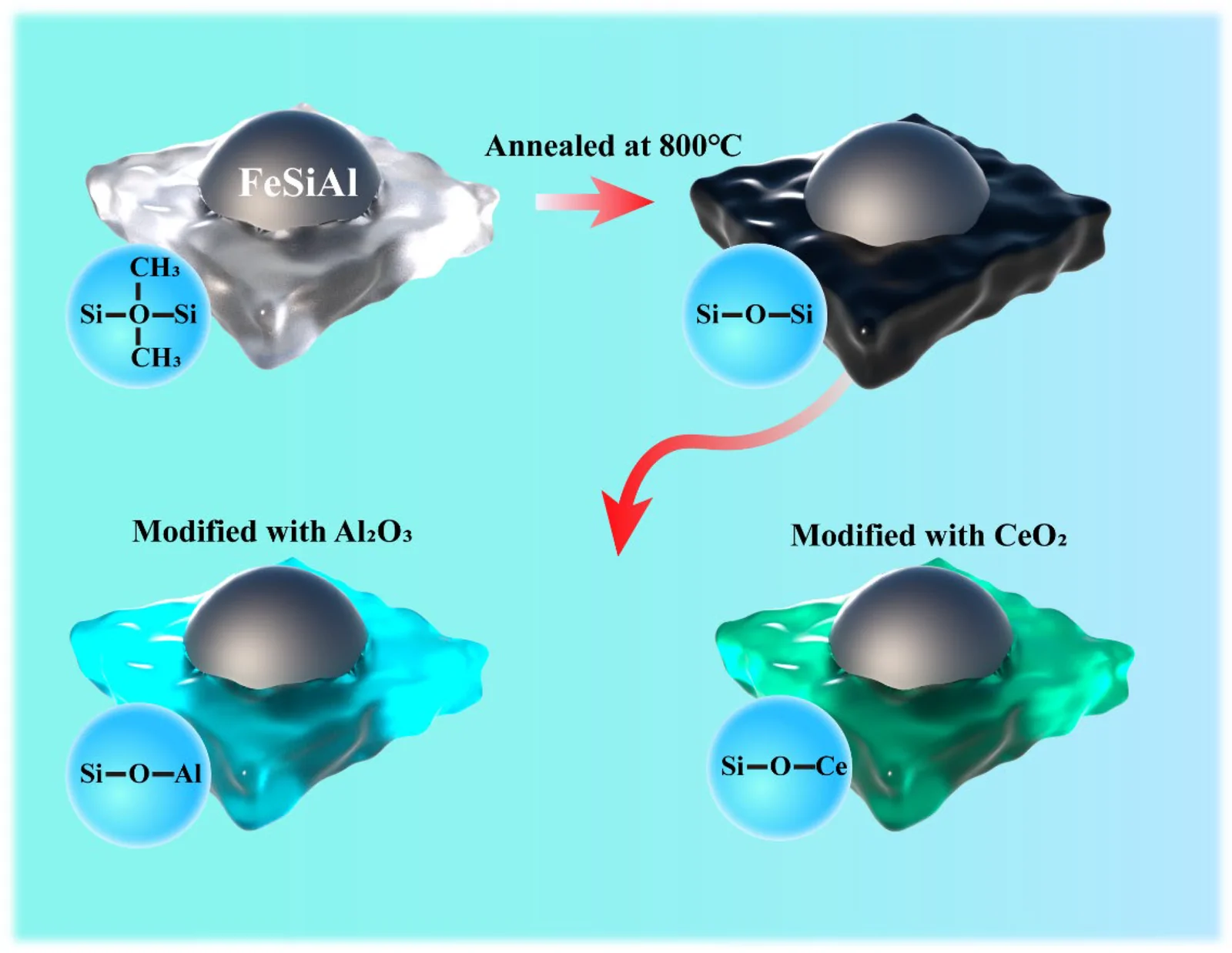
Schematic illustration of the high-temperature treatment and local coordination regulation of silicone-derived Si–O interfaces by Al_2_O_3_ and CeO_2_ in FeSiAl SMCs.

In this study, Al_2_O_3_ and CeO_2_ nano-oxides were introduced to regulate the silicone-resin-derived Si–O interface in FeSiAl SMCs, as schematically shown in Figure 10. The main conclusions are as follows:

(1) After annealing at 800 °C, the silicone resin forms an inorganic insulating interface dominated by an amorphous Si–O-rich network, which helps maintain interparticle electrical insulation. Together with residual-stress relaxation, this contributes to improved soft-magnetic performance.
(2) Al_2_O_3_ and CeO_2_ produce distinct interfacial spatial distributions and local chemical environments without altering the fundamental formation of the Si–O–Si network. Al_2_O_3_ is relatively uniformly distributed at an appropriate loading but tends to agglomerate at excessive addition, whereas CeO_2_ forms a denser and more continuous particulate interfacial distribution. Correspondingly, Si–O–Al/Al–O and Si–O–Ce/Ce–O-related interfacial environments are formed in the two systems.
(3) The different interfacial regulation modes lead to distinct magnetic responses. CeO_2_ modification is more favorable for maintaining high effective permeability, whereas Al_2_O_3_ provides stronger core-loss suppression. A0.5 achieves the lowest P_cv_ of 144.31 mW/cm^3^ at 100 kHz and 50 mT.
(4) Nano-oxide modification also alters the relative contributions of the fitted loss components. Si2A800 exhibits a redistribution of P_h_, P_e_, and P_ex_ with increasing frequency, while the fitted loss of A0.5 is dominated by P_ex_ and that of C0.5 shifts toward P_e_ dominance at higher frequencies.

## Figures and Tables

**Table 1:** Key magnetic properties of FeSiAl SMCs before and after annealing at different temperatures.

Samples	Sample Description	μ_e_ 100 kHz	DC (%) 100 Oe	P_cv_ (mW/cm^3^)	
				50 kHz/50 mT	100 kHz/50 mT
Si2	Unannealed	38.74	61.68	405.11	850.29
Si2A400	Annealed at 400 °C	42.41	58.40	331.58	715.14
Si2A500	Annealed at 500 °C	56.48	47.90	182.49	410.22
Si2A600	Annealed at 600 °C	70.79	41.00	78.037	202.36
Si2A700	Annealed at 700 °C	92.61	32.55	78.748	200.81
Si2A800	Annealed at 800 °C	97.39	32.74	71.975	183.53
Si2A900	Annealed at 900 °C	22.79	39.40	797.4	1552.4

**Table 2:** Key magnetic properties of silicone-resin-coated FeSiAl SMCs modified with different contents of Al_2_O_3_ and CeO_2_ after annealing at 800 °C.

Samples	Sample Description	μ_e_ 100 kHz	DC (%) 100 Oe	P_cv_ (mW/cm^3^)	
				50 kHz/50 mT	100 kHz/50 mT
A0.25	0.25 wt.% Al_2_O_3_	97.79	30.33	469.88	991.19
A0.5	0.5 wt.% Al_2_O_3_	91.74	33.52	54.411	144.31
A1	1 wt.% Al_2_O_3_	90.48	31.29	61.248	177.08
C0.25	0.25 wt.% CeO_2_	93.41	31.25	90.518	281.57
C0.5	0.5 wt.% CeO_2_	94.87	32.67	97.186	315.75
C1	1 wt.% CeO_2_	97.88	29.60	88.414	228.71

## Data Availability

The original contributions presented in this study are included in the article. Further inquiries can be directed to the corresponding authors.
